# *Coxiella burnetii* Induces Apoptosis during Early Stage Infection via a Caspase-Independent Pathway in Human Monocytic THP-1 Cells

**DOI:** 10.1371/journal.pone.0030841

**Published:** 2012-01-27

**Authors:** Yan Zhang, Guoquan Zhang, Laura R. Hendrix, Vernon L. Tesh, James E. Samuel

**Affiliations:** 1 Department of Microbial and Molecular Pathogenesis, College of Medicine, Texas A & M Health Science Center, Bryan, Texas, United States of America; 2 Department of Veterinary Pathobiology, College of Veterinary Medicine, University of Missouri-Columbia, Columbia, Missouri, United States of America; University of Louisville, United States of America

## Abstract

The ability of *Coxiella burnetii* to modulate host cell death may be a critical factor in disease development. In this study, human monocytic THP-1 cells were used to examine the ability of *C. burnetii* Nine Mile phase II (NMII) to modulate apoptotic signaling. Typical apoptotic cell morphological changes and DNA fragmentation were detected in NMII infected cells at an early stage of infection. FACS analysis using Annexin-V-PI double staining showed the induction of a significant number of apoptotic cells at an early stage of NMII infection. Double staining of apoptotic cell DNA and intracellular *C. burnetii* indicates that NMII infected cells undergoing apoptosis. Interestingly, caspase-3 was not cleaved in NMII infected cells and the caspase-inhibitor Z-VAD-fmk did not prevent NMII induced apoptosis. Surprisingly, the caspase-3 downstream substrate PARP was cleaved in NMII infected cells. These results suggest that NMII induces apoptosis during an early stage of infection through a caspase-independent pathway in THP-1 cells. In addition, NMII-infected monocytes were unable to prevent exogenous staurosporine-induced apoptotic death. Western blot analysis indicated that NMII infection induced the translocation of AIF from mitochondria into the nucleus. Cytochrome *c* release and cytosol-to-mitochondrial translocation of the pore-forming protein Bax in NMII infected cells occurred at 24 h post infection. These data suggest that NMII infection induced caspase-independent apoptosis through a mechanism involving cytochrome *c* release, cytosol-to-mitochondrial translocation of Bax and nuclear translocation of AIF in THP-1 monocytes. Furthermore, NMII infection increased TNF-α production and neutralization of TNF-α in NMII infected cells partially blocked PARP cleavage, suggesting TNF-α may play a role in the upstream signaling involved in NMII induced apoptosis. Antibiotic inhibition of *C. burnetii* RNA synthesis blocked NMII infection-induced PARP activation. These results suggest that both intracellular *C. burnetii* replication and secreted TNF-α contribute to NMII infection-triggered apoptosis during an early stage of infection.

## Introduction

*Coxiella burnetii* is an obligate intracellular Gram-negative bacterium that causes acute Q fever and chronic infections in humans [1]. Acute Q fever manifests as febrile illness, atypical pneumonia or hepatitis that usually is self-limiting or effectively treated by antibiotics. However, *C. burnetii* infections in immunocompromised persons, such as HIV or TB patients and pregnant women often develop into chronic disease [2]. Chronic Q fever is a severe, sometimes fatal disease and patients have very limited clinical benefit from various antibiotics regimens [1], [3]. The heart is the most frequently involved organ in patients with chronic Q fever and endocarditis is the most common manifestation. More importantly, the highly infectious nature of *C. burnetii* and its hardiness in adverse environmental conditions make the organism potentially useful in bioterrorism and biological warfare [4].

*C. burnetii* undergoes a phase variation where virulent phase I (PI) convert to avirulent phase II (PII) upon serial passage in a nonimmunologically competent host [5]–[7]. Smooth-LPS PI was able to replicate in wild type animals and cause disease in humans, while rough-LPS PII can be rapidly cleared in animals and does not cause disease in humans [8], [9]. Although *C. burnetii* can infect a wide range of host cells during the infection in humans and animals, the primary targets are monocytes/macrophages [1], [10]. It has been shown that both PI and PII organisms are able to proliferate within a large replication vacuole (LRV) in an acidic environment with a slow rate (doubling time of 20 h) of intracellular multiplication [1], [3], [11]–[13]. Others have shown that PI organisms survive within cells by escaping intracellular killing via prevention of early phagosome maturation, but PII organisms are rapidly killed by the phagolysosomal pathway [1], [14]–[16]. Accumulating evidence suggests that the mechanisms for *C. burnetii* intracellular survival and the establishment of a persistent infection may be related to its ability to modulate host responses and subvert microbiocidal functions of monocytes/macrophages [17]–[19].

Apoptosis is generally considered to be a host mechanism to remove damaged or abnormal cells without initiating an inflammatory response. The molecules of the apoptotic machinery are cysteine proteases (caspases) and the B-cell lymphoma protein-2 (Bcl-2) family of proteins [2]. Two main pathways have been shown to lead to host cell death. The intrinsic pathway is characterized by activation of Bcl-2 proteins resulting in increased mitochondrial membrane permeability and cytochrome *c* release into the cytoplasm, while the extrinsic pathway is mediated by the activation of initiator caspases following engagement of cell surface death receptors with their death-inducing ligands. Both the intrinsic and extrinsic pathways activate the executioner caspase, caspase-3, which in turn activates a number of downstream substrates involved in DNA fragmentation and changes in cell morphology. Apoptosis via a caspase-independent pathway has been described recently in cells infected with intracellular pathogens such as *Mycobacterium tuberculosis* and *Chlamydiales sp.*, demonstrating that, in the absence of caspase-3 activation, cells undergo apoptosis with the release of mitochondrial cytochrome *c* and apoptosis inducing factor (AIF) [2], [20], [21]. AIF nuclear translocation may be the result of the activation of Poly (ADP-ribose) polymerase (PARP), a family of proteins involved in regulating the mechanisms of caspase-independent cell death by reactive oxygen/nitrogen (ROS/RNS) species [22]–[24].

Apoptosis of infected cells has been demonstrated to be an important host defense mechanism to eliminate infected cells and block microbial dissemination [2], [25]–[28]. However, several obligate intracellular bacterial pathogens possess the ability to modulate host cell apoptotic signaling to facilitate their intracellular survival and dissemination. It has been shown that *Brucella suis* and *Rickettsia rickettsii* are able to inhibit apoptosis [2], [27], while *Legionella pneumophila* can induce apoptosis at a late stage of infection [29]. Modulation of the apoptotic pathways by *M. tuberculosis* and *Chlamydia sp.* may be more complex, as they appear to inhibit or induce apoptosis depending on their replication stage [2], [20], [30]. One recent study showed that *C. burnetii* was able to partially prevent exogenously-induced apoptosis in differentiated THP-1 cells as well as in primary monkey alveolar macrophages [31]. Similarly, *C. burnetii* PII organisms inhibited exogenously-induced apoptosis in Chinese hamster ovary and HeLa cells at a late infection stage [32]. These observations suggest that *C. burnetii* infected cells possess an anti-apoptosis ability in the presence of exogenously applied apoptotic stimuli that may be important for *C. burnetii* to establish a persistent infection. An earlier study showed TNF-α signaling is partially involved in interferon-γ (IFN-γ)-induced apoptosis in *C. burnetii* infected THP-1 monocytes [33].

However, since the impact of *C. burnetii* infection on host cell apoptosis at an earlier stage of infection is poorly understood, we tested the ability of *C. burnetii* to modulate host cell apoptotic signaling in human monocytic THP-1 cells. These results provide novel evidence to demonstrate that *C. burnetii* NMII induces apoptosis during an early stage of infection through a caspase-independent pathway.

## Materials and Methods

### *C. burnetii* isolates

C*oxiella burnetii* Nine Mile phase II RSA 439 (NMII) was used in this study [34]. NMII were cultured in L929 cells (ATCC) and were purified by gradient centrifugation as described elsewhere [35]. Purified bacteria were frozen at −80°C and used to infect Human myelogenous leukemia (THP-1) cells (ATCC) in this study.

### Cell culture and *C. burnetii* infection

THP-1 cells were cultured in 10% fetal bovine serum (FBS) supplemented RPMI 1640 medium (containing 100 U/ml penicillin and 100 µg/ml streptomycin) at 37°C in 5% CO_2_ in a humidified incubator. Before infection, 1×10^6^ THP-1 cells were cultured in antibiotic-free medium containing 5% FBS for at least 1 day and then infected with NMII at multiplicities of infection (MOI) of 5 and 50 or left uninfected. After incubation for 24 h, infected cells were washed twice with culture medium to remove extracellular bacteria and incubation was continued for different time points depending on the experiment.

### Indirect immunofluorescence assay (IFA)

Cells were infected with NMII at an MOI of 50 for 24 h. Infected cells were then washed and incubation was continued for 48 h. Infected and uninfected THP-1 cells seeded on glass coverslips were fixed with 2% paraformaldehyde for 15 min and then permeabilized with cold absolute methanol for 10 min. Intracellular *C. burnetii* were stained with rabbit anti-NMII polyclonal antibodies (1∶500), followed by incubation with goat anti-rabbit IgG (Invitrogen). Host cell nuclei were stained by Hoechst (1∶1000) and slides were examined using fluorescence microscopy.

### Phase-contrast microscopy

NMII infected and uninfected THP-1 cells were cultured in 24 well plates. At 48 h post infection, uninfected control cells were treated with staurosporine (1 µm) for 4 h as a positive control. Cellular morphological changes were observed using a Nikon Diaphot inverted phase-contrast microscope. Images were captured using a Dage Cooled Charge-Coupled Device Video camera and QED software.

### Quantitative real-time PCR

NMII infected and uninfected THP-1 cells were directly lysed with lysis buffer at 24–72 h post infection. DNA extraction and real-time PCR were performed as described previously [35]. Briefly, samples were pretreated with proteinase K, followed by DNA extraction using high Pure PCR Template Preparation Kit (Roche). DNA samples were used as templates to quantify the number of *C. burnetii com1* gene copies.

### DNA fragmentation assay

Cellular genomic DNA was extracted from NMII infected and uninfected THP-1 cells by using the Apoptotic DNA Ladder Kit according to the manufacturer's instructions (Roche Diagnostics Corp. Indianapolis, IN) at 24 h and 48 h post infection. DNA isolated from staurosporine (1 µM)-treated uninfected cells was used as a positive control. Equal amounts of DNA (2–4 µg) from different samples were run on 1.8% agarose gels and stained with ethidium bromide. DNA bands were photographed on the BioRad Gel Imager (Bio-Rad, Hercules, CA).

### Double staining of intracellular *C. burnetii* and apoptotic cell DNA

Monocytic THP-1 cells (1×10^6^) were seeded at collagen-coated coverslips with 2% FBS in antibiotic-free RPMI 1640 to increase adherence of monocytic cells. Cells were then infected with NMII at MOI 50 and were washed after 24 h post infection to remove extracellular *C. burnetii*. Infected cells were fixed and permeabilized at the end of each time point. Intracellular *C. burnetii* was stained with rabbit anti-NMII polyclonal antibodies followed by incubation with goat anti-rabbit IgG (Invitrogen). Apoptotic cell nuclei were stained with *in situ* cell death detection kit according to the manufacturer's instructions (Roche). Briefly, flowing washing, terminal deoxynucleotidyltransferase-mediated dUTP-biotin nick end labeling (TUNEL) reaction mix was added and incubated for 1 h at 37°C in the dark. Cells were then washed with PBS and examined using a fluorescence microscope.

### FACS analysis of apoptosis

Approximately 1×10^6^ NMII infected and unpinfected THP-1 cells were double stained with Annexin-V-FITC and propidium iodide (PI) at different time points post infection by using the Annexin-V-FLUOS Staining Kit according to the manufacturer's protocol (Roche). Following staining, the cells were washed twice with FACS buffer (0.5% BSA) and fixed with 1% paraformaldehyde for 15 min. Fluorescence was detected using a fluorescence-activated cell sorter (FACS Calibur, Becton-Dickinson, Palo Alto, CA) to analyze necrotic (PI+), non-apoptotic (negative for both dyes), early apoptotic (Annexin+/PI−) and late apoptotic cells (Annexin+/PI+). Fluorescence parameters were gated using unstained and single-stained uninfected control cells and 10,000 cells were counted for each sample.

### Western blot analysis of PARP and caspase-3

Cell extracts were prepared using M-PER® Mammalian Protein Extraction Reagent (Pierce, Rockford, IL) and protein concentrations were measured by using the Micro BCA protein assay Kit (Pierce, Rockford, IL). Equal amounts of proteins (50 µg) were separated by SDS-PAGE with 10% polyacrylamide gels and then transferred onto nitrocellulose membranes. Caspase-3-specific antibodies (Cell Signaling Technology, Danvers, MA) that recognize both the pro-form (37 kD) and a cleaved fragment (19 kD) were used to determine caspase-3 activity. PARP cleavage was detected by Western blotting using PARP-specific antibodies that recognize full length PARP (116 kD) and a cleaved PARP (89 kD), respectively. Reaction bands were visualized by using the Western Lightning chemiluminescence system (NEN-Perking Elmer, Boston, MA).

### Induction of apoptosis

To induce cell apoptosis, NMII infected and uninfected control cells were cultured in 24-well plates and treated with staurosporine (1 µM) for 4 h at 24 or 48 h post infection. Western blot and Annexin-V-PI staining by FACS analysis were used to detect the ability of NMII to prevent apoptosis in infected cells.

### Caspase inhibition

To inhibit caspase activity, NMII infected and uninfected THP-1 cells were cultured with a general caspase inhibitor, N-benzyl-oxycarbonyl-Val-Ala-Asp (OMe)-fluoromethylketone (ZVAD-fmk) (Calbiochem, San Diego, CA) at 50 µM for different time points post infection. In addition, uninfected THP-1 cells and staurosporine treated THP-1 cells were used as negative and positive controls, respectively.

### Preparation of mitochondria and nuclear fractions

Mitochondria and nuclear protein were extracted using Mitochondria Isolation Kit for Mammalian Cells and NE-PER Nuclear and Cytoplasmic Extraction Reagent (Pierce, Rockford, IL) respectively, following the protocols from the manufacturer. The pellet containing mitochondria was resuspended in 2% CHAPS in Tris buffered saline (TBS). Protease inhibitors (Pierce, Rockford, IL) were used according to manufacturer's instructions. Protein concentrations were measured using the Micro BCA protein assay Kit (Pierce, Rockford, IL).

### AIF nuclear translocation

NMII (MOI 5, 50) infected and uninfected cells were harvested and washed twice with PBS at 24 or 48 h post infection. Mitochondria and nuclear proteins were prepared. Equal amounts of proteins (50 µg) were separated by SDS-PAGE with 12% polyacrylamide gels and transferred to nitrocellulose membranes. After blocking in 5% non-fat dried milk at room temperature for 1 hour, membranes were probed with rabbit polyclonal anti-AIF antibody (Cell Signaling Technology Inc., MA). Reaction bands were visualized using the Western Lightning chemiluminescence system.

### Cytochrome *c* release and Bax translocation

NMII (MOI 5, 50) infected and uninfected cells were cultured in 24-well plates. Cells were harvested at 24 or 48 h post infection by using Mitochondria Isolation Kit for Mammalian Cells (Pierce, Rockford, IL) following the manufacturer's instructions. The mitochondrial pellet was resuspensed in 2% CHAPS in Tris buffered saline (TBS) with protease inhibitors and boiled with SDS-PAGE sample buffer for Western blotting. Equal amounts of proteins (50 µg) were separated by SDS-PAGE with 12% acrylamide gels and then transferred to nitrocellulose membranes. Membranes were probed with rabbit polyclonal anti-cytochrome *c* and anti-Bax antibody (Cell Signaling Technology Inc., MA). Reactive bands were visualized by using the Western Lightning chemiluminescence system.

### Neutralization of TNF-α and ELISA

Monocolonal anti-human TNF-α/TNFSF1A antibody (R&D Systems, Minneapolis, MN) was used to neutralize secreted TNF-α in NMII infected cells. 1×10^6^ THP-1 cells were cultured in 24 well plates. Cells were then either infected with NMII (MOI 5 or 50) or treated with LPS (100 ng/ml) as a positive control. Anti-TNF-α antibody (0.05 µg/ml) was added following infection or LPS treatment. Supernatants were collected at 24, 48 and 72 h post infection. The TNF-α concentration in supernatants was measured by using Quantikine Immunoassay for Human TNF-α/TNFSF1A (R&D Systems) according to the manufacturer's protocol. The absorbance was read on an automated plate reader (Tecan U.S. Inc.) at 450 nm (reference wavelength, 650 nm). The assay was repeated three times and mean cytokine values ± SEM were represented as pg/ml.

### Antibiotic inhibition

To inhibit bacterial replication, 10 µg/ml of rifampin was added to cell culture medium along with the *C. burnetii* infection. Total DNA was extracted at 24, 48 and 72 h post infection and real-time PCR was used to evaluate the effectiveness of antibiotic inhibition. Western blotting was then performed to detect PARP cleavage.

### RNA isolation and cDNA preparation

Total cellular RNA was isolated from NMII infected and uninfected THP-1 cells by the RNeasy Mini Kit according to the manufacturer's instructions (Qiagen, MD) at 24 h and 48 h post infection, respectively. Staurosporine treated uninfected cells were used as apoptosis positive controls. cDNA was synthesized using QuantiTec Rev. Transcription Kit according to the manufacturer's instructions (Qiangen, MD).

### RT^2^ qPCR

Reverse transcription PCR (RT-PCR) was performed on cDNA using RT^2^ SYBR Green PCR Master Mix (AB Applied Biosystems, CA). Primers for human PARP and GAPDH were purchased from SuperArray (Frederick, MD). The results were analyzed by the recommended Δ Δ Ct Methods from SuperArray. GAPDH primers were used as a molecular control.

### Statistical analysis

A two-tailed Student's *t* test was used to compare the significance of apoptotic cells between NMII infected and uninfected THP-1 cells. *P* values<0.05 were considered significant.

## Results

### Intracellular infection of THP-1 cells with *C. burnetii*

**Figure 1:**
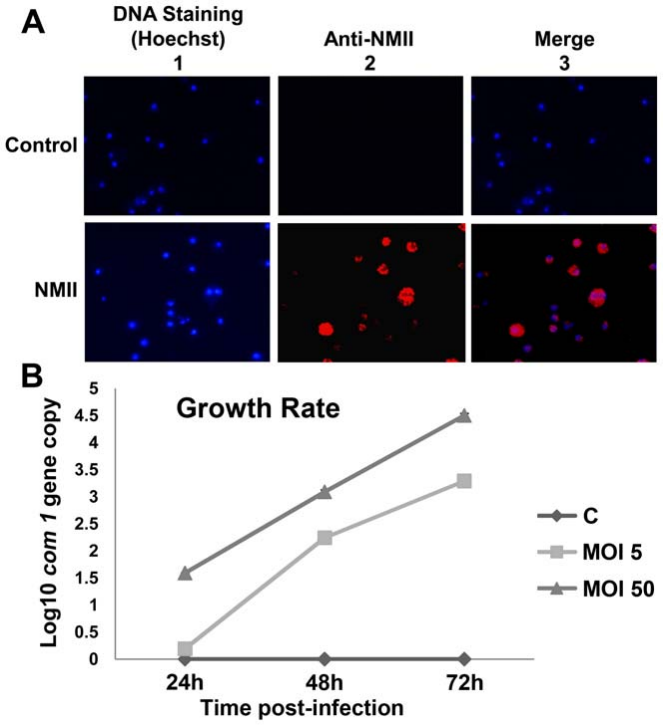
Growth rate and indirect immunofluorescence assay (IFA) staining of NMII in THP-1 cells. Cells were infected with NMII at MOI of 50 for 24 h. Infected cells were then washed and incubation was continued for 48 h. Infected and uninfected THP-1 cells were fixed with paraformaldehyde and permeabilized. Intracellular *C. burnetii* were stained by IFA with rabbit anti-*Coxiella* polyclonal antibodies and viewed using a fluorescence microscope. Panel 1A, IFA staining of NMII infected THP-1 cells. Upper panel: uninfected control cells; lower panel: NMII infected cells. 1 Hoechst staining for host cell DNA; 2 Cells stained with anti-*Coxiella* antibodies; and 3 Merge. Panel 1B, growth rate of NMII in THP-1 cells. NMII infected and uninfected monocytic THP-1 cells were directly lysed at 24, 48 or 72 h post infection. DNA was extracted and used as a template to quantify the number of *C. burnetii com1* gene copies by real-time PCR.

IFA was used to confirm intracellular *C. burnetii* infection in THP-1 cells. At 72 h post infection, control cells and *C. burnetii* infected cells were stained by IFA with rabbit anti-NMII polyclonal antibodies and viewed by fluorescence microscopy (Fig. 1A). The results indicated that NMII were able to infect THP-1 cells. Fig. 1B shows the growth rates of NMII at different times post infection with MOI 5 or 50 obtained by measuring *C. burnetii com1* gene copy numbers using a real-time PCR assay. The results indicate that numbers of *C. burnetii* increased after infection in a dose and time dependent manner.

### Morphological changes in NMII infected THP-1 cells

**Figure 2:**
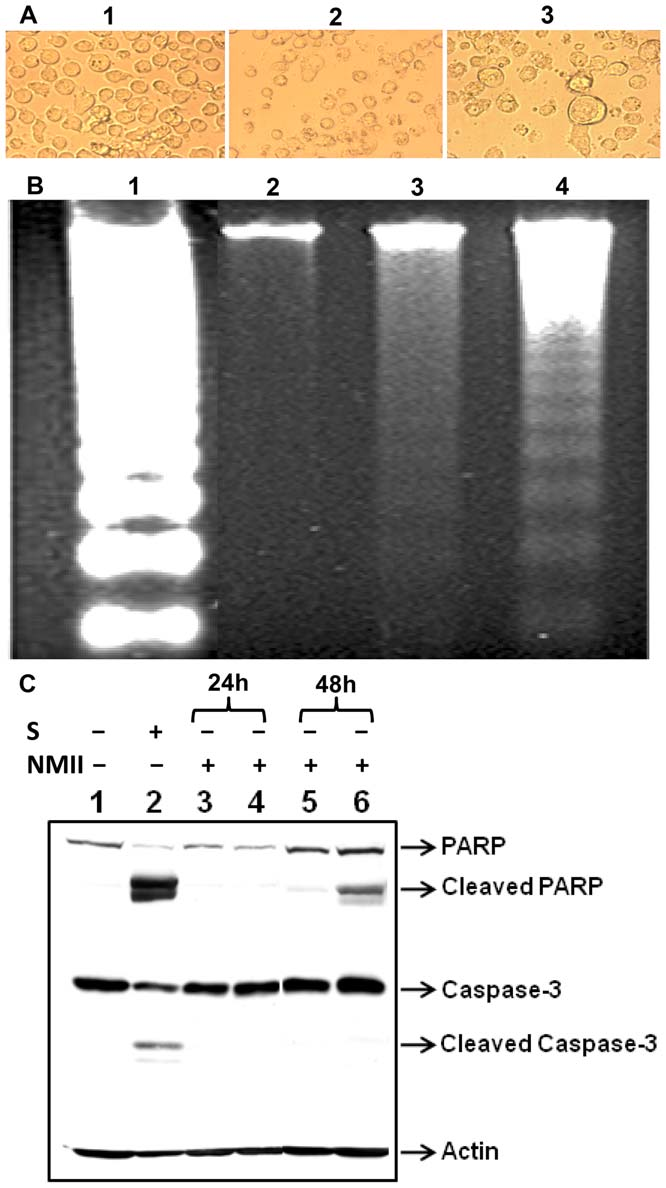
*C. burnetii* NMII infection induced cell death in THP-1 cells. NMII (MOI 50) infected and control cells were cultured in 24 well plates. Uninfected control cells were treated with staurosporine (1 µm) for 4 h. Panel 2A, morphological changes in NMII infected THP-1 cell at 48 h post infection. 1, control cells; 2, normal cells with staurosporine-treatment; and 3, NMII infected cells. Typical apoptotic morphological changes were displayed in staurosporine-treated and NMII infected cells. Arrow indicates large vacuoles that contain intracellular organisms in NMII infected cells. Panel 2B, pattern of DNA fragmentation during NMII infection. Genomic DNA from NMII infected and control cells were extracted at 48 h post infection. DNA was separated on 1.8% agarose gels and stained with ethidium bromide. Lane 1, U937 apoptotic cells as positive control; Lane 2, Uninfected THP-1 cell control; Lane 3, NMII infected THP-1 cells; Lane 4, Staurosporine treated THP-1 cells. Panel 2C, Western blot of PARP and caspase-3 activities in NMII induced apoptosis. Lane 1, normal cells; Lane 2, staurosporine treated positive control cells; Lane 3, NMII (MOI 5) infected cells at 24 h post infection; Lane 4, NMII (MOI 50) infected cells at 24 h post infection; Lane 5, NMII (MOI 5) infected cells at 48 h post infection; Lane 6, NMII (MOI 50) infected cells at 48 h post infection.

Cell membrane blebbing and shrinking are typical characteristics of apoptotic cells [36]. To determine if *C. burnetii* infection is able to induce apoptosis, THP-1 cells were infected with NMII at an MOI of 50. An apoptosis-inducer, staurosporine, was used as a positive control for comparison to morphological changes in NMII infected cells. Typical apoptotic morphological changes such as cell shrinkage and breakdown to small apoptotic bodies were seen in staurosporine-treated cells. Similar morphology changes were also observed in NMII infected cells at 48 h post infection (Fig. 2A). Large vacuoles that contain intracellular NMII were also observed in NMII infected THP-1 cells. These results indicate that NMII may induce apoptosis in THP-1 cells at an early stage post infection.

### DNA fragmentation of NMII infected THP-1 cells

Inter-nucleosomal degradation of nuclear DNA into multimers of 180–200 bp is one of the hallmarks of apoptotic cell death [37]. DNA fragmentation was analyzed in NMII infected THP-1 cells and used as an additional indicator to determine if NMII infection induces host cell apoptosis. THP-1 cells were infected with NMII at MOI 50 for 24 h. Cellular genomic DNA was isolated from NMII infected cells and uninfected cells at 72 h post infection. DNA fragmentation was observed in both staurosporine-treated and in NMII infected cells compared to uninfected control cells (Fig. 2B). This result supports the interpretation that NMII infection can induce apoptosis in THP-1 cells during an early stage of infection.

### Caspase-3 and PARP activities in NMII induced apoptosis

To characterize the apoptotic cell signaling pathway induced by NMII, activation of the apoptosis executioner, caspase-3, and its downstream substrate PARP, were examined in NMII infected THP-1 cells by Western blotting. Staurosporine-treated uninfected cells were used as a positive control. A cleaved PARP fragment was detected at 24 and 48 h post infection (Fig. 2C) in NMII infected cells. However, caspase-3 was not cleaved in NMII infected cells as compared to the control cells. In addition, a reduction in PARP mRNA level was also confirmed by reverse transcription quantitative real time PCR in NMII infected cells (data not shown). These results indicate that PARP activated in NMII induced apoptosis in THP-1 cells, but caspase-3 did not activate, suggesting that NMII induced apoptosis may occur through a caspase-independent apoptotic signaling pathway.

### NMII infection induced significant high level of apoptotic cells

**Figure 3:**
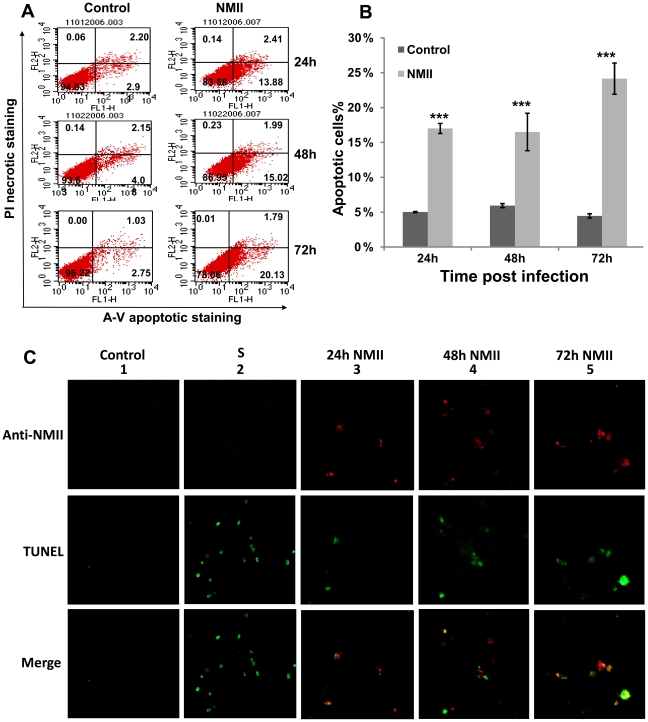
FACS Analysis of Annexin-V staining of NMII infected THP-1 cells. Approximately 1×10^6^ NMII infected or uninfected cells were double stained with Annexin-V-FITC and PI. Panel 3A, representative FACS scatter plots of THP-1 cells. Fluorescence was detected using a fluorescence-activated cell sorter to analyze necrotic (PI+), non-apoptotic (negative for both dyes), early apoptotic (Annexin+/PI−), and late apoptotic cells (Annexin+/PI+). Panel 3B, percentages of apoptotic cells in NMII infected THP-1 cells. Data shown represents the Mean±SE from at least three independent experiments. ^*^denotes significant differences (***p<0.001) between infected and uninfected cells at each time point post infection. Panel 3C, double staining of intracellular *C. burnetii* and apoptotic cell DNA. Intracellular *C. burnetii* was stained by IFA with rabbit anti-*Coxiella* polyclonal antibodies and apoptotic host nuclei were stained with TUNEL staining. Upper panel: Cells stained with anti-*Coxiella* antibodies; Middle panel: TUNEL stating of apoptotic host nuclei; Lower panel: Merge. From left to right 1) Normal cell control; 2) Staurosporine (1 µm) treated apoptotic control cell; 3) NMII infected cells at 24 h post infection; 4) NMII infected cells at 48 h post infection; 5) NMII infected cells at 72 h post infection.

Disruption of phospholipid asymmetry and exposure of phosphatidylserine (PS) on the outer surface of the plasma membrane are well established early markers of apoptosis and may be detected by FACS analysis with Annexin-V staining [36], [37]. To quantitate the NMII induced apoptotic cell death in THP-1 cells, approximately 1×10^6^ NMII infected and uninfected cells were double stained with Annexin-V-FITC and propidium iodide (PI) at different times post infection. Apoptotic cell death was detected from 24 h post infection in NMII infected cells (Fig. 3A). FACS analysis identified significantly higher numbers of apoptotic cells in NMII infected cells than uninfected control cells at an early stage of infection (Fig. 3B). The FACS analysis provided strong support to the interpretation that NMII can induce apoptosis in THP-1 cells during an early stage of infection.

### Apoptotic cell DNA appeared in *C. burnetii* infected THP-1 cells

In order to determine whether *C. burnetii* infected cells or bystander cells are undergoing apoptosis, NMII infected THP-1 cells were double stained for intracellular *C. burnetii* and apoptotic cell nuclei by IFA at 24, 48 and 72 h post infection. As showed in Figure 3C, compared to the uninfected control cells, TUNEL staining positive cells appeared in some NMII infected cells at 24 h post infection and apoptotic cell number increased at 48 h and 72 h post infection in a time dependent manner. Interestingly, colocalization between apoptotic cell DNA and intracellular *C. burnetii* was observed in *C. burnetii* infected cells at different time points post infection. These results provided direct evidence to demonstrate that NMII infected THP-1 cells undergoing apoptosis.

### A caspase-independent apoptotic signaling pathway is involved in NMII induced apoptosis

**Figure 4:**
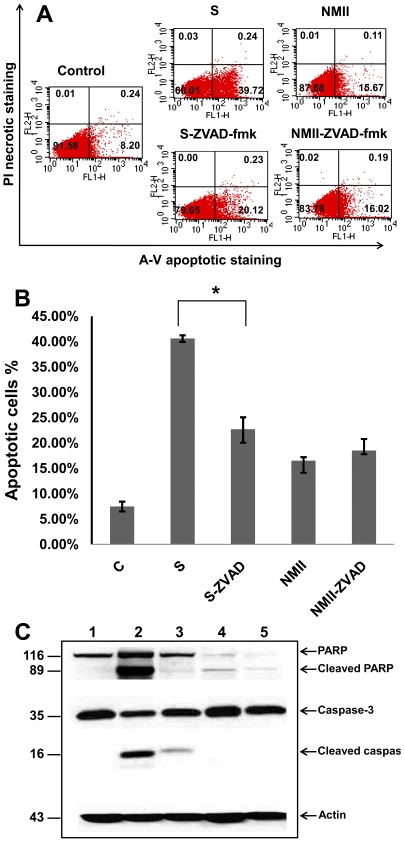
The caspase inhibitor ZVAD-fmk failed to inhibit NMII induced cell death. Panel 4A, FACS analysis of Annexin-V staining in ZVAD-fmk treated NMII infected THP-1 cells. Control cells were treated with staurosporine (1 µm) or staurosporine with ZVAD-fmk (50 µm) for 4 h. NMII infected THP-1 cells were treated with ZVAD-fmk (50 µm) and refreshed daily up to 48 h post infection. Fluorescence was detected using a fluorescence-activated cell sorter to analyze necrotic (PI+), non-apoptotic (negative for both dyes), early apoptotic (Annexin+/PI−), and late apoptotic cells (Annexin+/PI+). Panel 4B, percentage of apoptotic cells in Z-VAD-fmk treated NMII infected THP-1 cells. Data shown are the Mean±SE from at least three independent experiments. ^*^denotes significant differences (*p<0.05). Panel 4C, Western blot of Caspase-3 and PARP activities in ZVAD-fmk treated NMII infected cells. Lane 1, normal THP-1 cells; lane 2, staurosporine treated THP-1 cells; lane 3, staurosporine with ZVAD-fmk treated THP-1 cells; lane 4, NMII infected THP-1 cells; lane 5, NMII infected THP-1 cells treated with ZVAD-fmk.

To further confirm if NMII induces caspase-independent apoptotic cell death, a general caspase inhibitor ZVAD-fmk was used to inhibit caspase activity in staurosporine treated uninfected control cells and NMII infected cells. The inhibition of apoptotic cell death by ZVAD-fmk was evaluated by FACS analysis with Annexin-V staining (Fig. 4A). FACS analysis indicated ZVAD-fmk treatment significantly reduced staurosporine-induced apoptotic cells but there was no difference in numbers of apoptotic cells between ZVAD-fmk treated and untreated NMII infected cells (Fig. 4B). This result demonstrates that ZVAD-fmk inhibits staurosporine-induced apoptosis but was unable to inhibit NMII induced apoptosis. To know whether ZVAD-fmk treatment can affect caspase-3 and PARP activities in NMII infected THP-1 cells, cleaved caspase-3 and PARP were also detected by Western blot. Compared to staurosporine only treated THP-1 cells, both cleaved caspase-3 and PARP were decreased in ZVAD-fmk and staurosporine treated cells (Fig. 4C). These results demonstrate that ZVAD-fmk treatment inhibits staurosporine-induced caspase-3 and PARP activation. However, there was no observable difference in cleaved PARP between ZVAD-fmk treated and untreated NMII infected cells and no cleaved caspase-3 was detected in NMII infected cells. These results indicate that ZVAD-fmk treatment fails to inhibit NMII induced apoptosis and support the interpretation that NMII induces caspase-independent, PARP-mediated apoptosis in monocytic THP-1 cells.

### NMII infected cells are unable to resist of exogenously induced apoptosis

**Figure 5:**
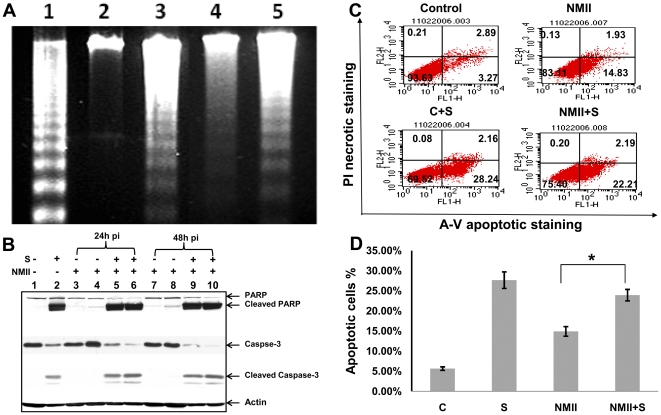
Inability of *C. burnetii* infection to inhibit exogenously induced apoptosis. THP-1 cells were infected with NMII at MOI 5 or 50 and left untreated or treated with staurosporine (1 µm) for 4 h. Panel 5A, genomic DNA from 48 h post infected cells and control cells were run on 1.8% agarose gels and stained with ethidium bromide. Lane 1, U937 apoptotic cells as positive control; Lane 2, Uninfected THP-1 cell control; Lane 3, THP-1 cells treated with 1 µm staurosporine for 4 h. Lane 4, NMII infected THP-1 cells; Lane 5, NMII infected cells treated with 1 µm staurosporine for 4 h. Panel 5B, FACS Analysis of staurosporine-treated NMII infected THP-1 cells. Fluorescence was detected using a fluorescence-activated cell sorter to analyze necrotic (PI+), non-apoptotic (negative for both dyes), early apoptotic (Annexin+/PI−), and late apoptotic cells (Annexin+/PI+). Panel 5C, percentage of apoptotic cells in staurosporine treated NMII infected THP-1 cells. Data shown are the Mean±SE from at least three independent experiments. ^*^denotes significant differences (*p<0.05). Panel 5D, Western blot of caspase-3 and PARP activities in staurosporine treated NMII infected THP-1 cells. Lane 1, normal cells; Lane 2, staurosporine treated cells; Lane 3, NMII (MOI 5) infected cells at 24 h post infection; Lane 4, NMII (MOI 50) infected cells at 24 h post infection; Lane 5, staurosporine treated NMII (MOI 5) infected cells at 24 h post infection; Lane 6, staurosporine treated NMII (MOI 50) infected cells at 24 h post infection; Lane 7, NMII (MOI 5) infected cells at 48 h post infection; Lane 8, NMII (MOI 50) infected cells at 48 h post infection; Lane 9, staurosporine treated NMII (MOI 5) infected cells at 48 h post infection; Lane 10, staurosporine treated NMII (MOI 50) infected cells at 48 h post infection.

Previous studies have shown that *C. burnetii* infected cells possess the ability to inhibit apoptosis induced by exogenously administered inducers [31], [32]. To determine the ability of NMII to prevent host cell apoptosis, DNA fragmentation (Fig. 5A) and Annexin-V-PI staining by FACS analysis (Fig. 5B) were performed to test whether NMII can inhibit apoptosis induced with staurosporine (1 µM) in THP-1 cells. Results show NMII infection is unable to inhibit staurosporine-induced DNA fragmentation. FACS analysis indicates apoptotic cell numbers were not significantly different between uninfected control cells and NMII infected cells 4 h after staurosporine treatment (Fig. 5C). These results were also confirmed at the protein level by Western blot (Fig. 5D). Both caspase-3 and PARP were cleaved in staurosporine treated infected cells similar to the positive control cells from 24 to 48 h post infection. These results indicate that NMII was unable to prevent exogenously induced apoptotic death in monocytic THP-1 cells.

### AIF nuclear translocation

**Figure 6:**
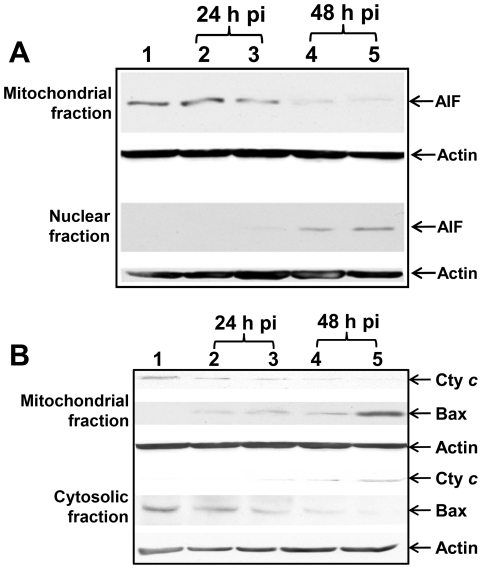
NMII induced apoptosis involved in AIF nuclear translocation and changes in mitochondrial membrane permeability. Panel 6A, Western blot of AIF translocation in NMII infected THP-1 cells. Infected cells were harvested at different time points post infection. Mitochondrial or nuclear fraction was prepared for analysis of AIF translocation. Lane 1, control cells; lane 2, NMII (MOI 5) infected cells at 24 h post infection; lane 3, NMII (MOI 50) infected cells at 24 h post infection; lane 4, NMII (MOI 5) infected cells at 48 h post infection; lane 5, NMII (MOI 50) infected cells at 48 h post infection. Panel 6B, Western blot analysis of cytochrome *c* release and translocation of Bax. Lane 1, control cells; lane 2, NMII (MOI 5) infected cells at 24 h post infection; lane 3, NMII (MOI 50) infected cells at 24 h post infection; lane 4, NMII (MOI 5) infected cells at 48 h post infection; lane 5, NMII (MOI 50) infected cells at 48 h post infection.

Mitochondrial protein AIF has been shown to play an important role in caspase-independent apoptosis. PARP has been reported to be an AIF- releasing factor [23]. To identify downstream signaling in PARP-mediated caspase-independent apoptosis, we investigated the activity of mitochondrial protein AIF in NMII infected cells. Western blots showed that NMII (MOI 5, 50) infection induced translocation of AIF into the nucleus (Fig. 6A) from 24 to 48 h post infection. This result suggests that NMII induced caspase-independent apoptosis may act through a mechanism involving nuclear translocation of AIF in monocytic THP-1 cells.

### Cytochrome *c* release and Bax translocation

The induction of changes in mitochondrial membrane permeability in NMII infected cells were further analyzed by detecting release of cytochrome *c* and the cytosol-to-mitochondrial translocation of the pore forming protein Bax. Western blot analysis revealed that cytochrome *c* was detected in the cytosolic fraction and was decreased in the mitochondrial fraction from 24 to 48 h post infection (Fig. 6B). In contrast, Bax was increased in the mitochondrial fraction but was less detectable in the cytosolic fraction at 24 and 48 h post infection. It is known that the cytosol-to-mitochondrial translocation of Bax leads to the loss of mitochondrial membrane potential, resulting in cytochrome *c* release in apoptotic cells. These results provide strong evidence to demonstrate that NMII induced caspase-independent apoptosis at an early infection stage is mediated by both Bax translocation and release of cytochrome *c*.

### Neutralization of secreted TNF-α partially blocked PARP cleavage

**Figure 7:**
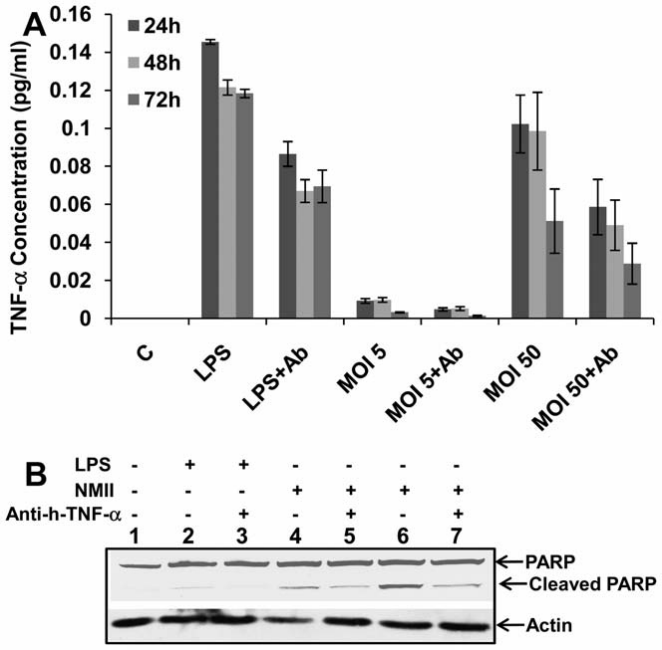
Neutralization of secreted TNF-α partially blocked PARP cleavage. Panel 7A, concentration of TNF-α in NMII infected THP-1 cell supernatants as measured by ELISA. LPS treated THP-1 cells were used as positive controls. THP-1 cells were infected with different MOIs (5 or 50) of NMII. LPS (100 ng/ml) treated or NMII infected THP-1 cells were treated with anti-human TNF-α/TNFSF1A (0.05 µg/ml). Supernatants were collected at 24, 48 and 72 h post-treatment or infection, respectively. Panel 7B, Western blot of PARP activity in TNF-α neutralized NMII infected THP-1 cells. Lane 1, control cells; lane 2, LPS treated cells at 48 h; lane 3, LPS treated cells with anti-human TNF-α at 48 h; lane 4, NMII (MOI 5) infected cells at 48 h post infection; lane 5, NMII (MOI 5) infected cells with anti-human TNF-α at 48 h post infection; lane 6, NMII (MOI 50) infected cells at 48 h post infection; lane 7, NMII (MOI 50) infected cells with anti-human TNF-α at 48 h post infection.

PARP activation is an extremely sensitive indicator of DNA damage that may be induced by oxidative stress via TNF-α signaling [38]. Recent studies have shown that TNF-α is involved in both caspase-dependent and -independent apoptotic pathways [38], [39]. TNF signaling can also stimulate caspase-independent death through mitochondrial oxidative stress [38], [39]. Previous studies have shown NMII infected cells have upregulated TNF-α [19], [31], [40]. To further investigate upstream signals of the possible involvement of TNF-α in NMII induced caspase-independent apoptosis, we measured TNF-α release upon infection. A monocolonal anti-human TNF-α/TNFSF1A antibody was used to neutralize soluble TNF-α in the supernatants of infected cells. ELISA data indicated that the TNF-α concentration was increased in supernatants from high MOI NMII infected cells compared to control cells (Fig. 7A), while only a low level of TNF-α was detected in supernatants of low MOI (MOI 5) infected cells. Neutralization of secreted TNF-α reduced the concentration of TNF-α in the supernatants. Western blot analysis revealed that neutralization of TNF-α partially blocked PARP cleavage in NMII infected cells compared to the LPS treated control cells (Fig. 7B). Although with a very low level of TNF-α in the supernatants from NMII (MOI 5) infected cells, PARP cleavage was detected. These results suggest that TNF-α may be one factor involved in NMII induced caspase-independent apoptosis. Other signaling pathways or factors activated during infection may contribute to NMII induced apoptosis.

### NMII induced apoptosis depends on bacterial replication

**Figure 8:**
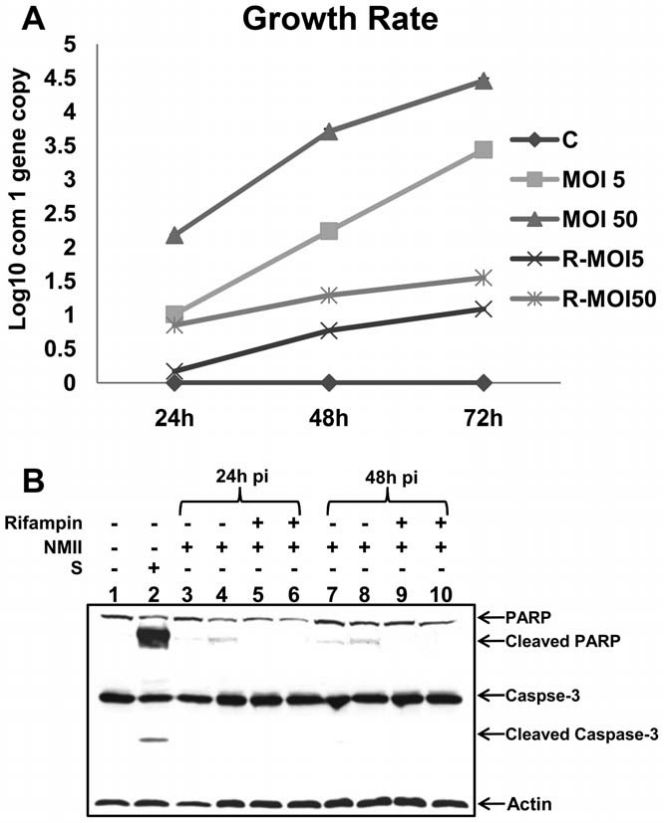
NMII induced apoptosis depends on *C. burnetii* replication. NMII infected THP-1 cells were treated with 10 µg/ml rifampin up to 72 h post infection. Panel 8A, quantification of *C. burnetii com1* gene copies by real-time PCR. Panel 8B, Western blot of rifampin inhibition in NMII infected THP-1 cells. Lane 1, normal cells; Lane 2, staurosporine treated positive control cells; Lane 3, NMII (MOI 5) infected cells at 24 h post infection; Lane 4, NMII (MOI 50) infected cells at 24 h post infection; Lane 5, NMII (MOI 5) infected cells with rifampin treatment at 24 h post infection; Lane 6, NMII (MOI 50) infected cells with rifampin treatment at 24 h post infection; Lane 7, NMII (MOI 5) infected cells at 48 h post infection; lane 8, NMII (MOI 50) infected cells at 48 h post infection; Lane 9, NMII (MOI 5) infected cells with rifampin at 48 h post infection; Lane 10, NMII (MOI 50) infected cells with rifampin treatment at 48 h post infection.

In order to understand whether bacterial replication is responsible for NMII induced apoptosis in THP-1 cells, we examined if inhibition of *C. burnetii* replication could block NMII induced apoptotic signaling. Since rifampin has been demonstrated to be the most effective antibiotic to inhibit *C. burnetii* replication [35], we used 10 µg/ml of rifampin to inhibit *C. burnetii* replication in NMII infected THP-1 cells. *C. burnetii* replication was monitored by measuring *C. burnetii com1* gene copies using real-time PCR. As shown in Fig. 8A, compared to rifampin untreated NMII infected cells, *C. burnetii* replication was significantly decreased by rifampin treatment at different times post infection. Interestingly, Western blot analysis indicated that cleaved PARP was observed in rifampin untreated NMII infected cells, but was undetectable in rifampin treated NMII infected cells at 24 and 48 h post infection (Fig. 8B). These results suggest that *C. burnetii* replication is required for NMII induced apoptosis in THP-1 cells.

## Discussion

Host cell death is one of the most critical determinants of the progression and outcome of diseases during microbial infections. Apoptosis of infected cells is an important defense mechanism of the mammalian host to limit microbial infections in an immunologically silent manner. Although accumulating evidence has demonstrated that several intracellular bacterial pathogens modulate host cell apoptotic signaling pathways to facilitate their intracellular survival, the relevance of modulating the host cell apoptotic signaling to bacterial pathogenesis and to the host immune response against intracellular pathogens remains unclear. Because C. *burnetii* is an obligate intracellular bacterial pathogen and its replication in the infected host depends on viable cells, the ability of *C. burnetii* to modulate host cell death may be a critical pathogenic factor in disease development. In order to understand whether *C. burnetii* can modulate host cell death, human monocytic THP-1 cells were used to examine the ability of *C. burnetii* NMII to modulate host cell apoptotic signaling. A typical apoptotic cell morphological change was observed in NMII infected cells and apoptosis was confirmed by DNA fragmentation and PARP cleavage in NMII infected cells in a dose dependent manner during an early stage of infection. To further characterize the ability of *C. burnetii* NMII to induce apoptosis, we examined the impact of *C. burnetii* NMII infection on the viability of THP-1 cells. FACS analysis of Annexin-V-PI double staining identified a significantly higher number of apoptotic cells in NMII infected THP-1 cells after 24 h post infection. Compared to the uninfected control cells, there were 15–20% apoptotic cells in the total viable cell population in NMII infected THP-1 cells. This level of apoptosis was maintained during the early stage infection. In addition, double staining of apoptotic cell DNA and intracellular *C. burnetii* indicates that NMII infection induced apoptosis occurred in *C. burnetii* infected cells. This is the first evidence to demonstrate that *C. burnetii* NMII is able to actively induce host cell apoptosis.

In order to identify the signaling pathway that is involved in NMII induced apoptosis, the activities of caspase-3 and its substrate PARP were examined in NMII infected monocytes. Interestingly, the results indicated that the caspase-3 was not activated, but its target molecule PARP, was activated during NMII infection. In addition, a pan caspase inhibitor, ZVAD-fmk, did not inhibit infection-induced apoptosis. These results indicate that caspase-3 does not participate in activated PARP-dependent cell death in NMII infected THP-1 cells. Cleaved caspase-3 was undetectable, suggesting that NMII induced apoptosis through a caspase-3-independent apoptotic pathway and that PARP activation may be critical for NMII induced apoptosis. This interpretation was supported by the observations that other intracellular bacterial pathogens, such as *M. tuberculosis* and *Chlamydia sp.*, were able to induce host cell apoptosis via a caspase-independent pathway [20], [21], [30]. Thus, a caspase-independent pathway may be uniquely utilized by some intracellular bacterial pathogens to modulate host cell death.

Previous studies have shown that *C. burnetii* infected cells possess the ability to inhibit apoptosis induced by exogenously administered inducers in differentiated THP-1 cells and monkey primary alveolar macrophages [31]. However, we did not observe resistance to staurosporine-induced caspase-dependent apoptosis as caspase-3 and its substrates PARP were also cleaved in infected monocytes in the presence of staurosporine. Since differentiated macrophage-like THP-1 cells display different biological functions in many aspects compared to undifferentiated monocytes [41]–[43]. It has been demonstrated differences in the kinetics and extent of apoptosis induction between monocytic and macrophage-like cells, with differentiation being associated with increased resistance to apoptosis [44], [45]. Thus, the sensitivity of host cells to apoptosis in response to bacterial stimuli may be varied based on the cell type, state of cellular maturation, and differentially expressed cell surface receptors. The different results between the studies suggest that *C. burnetii* may possess the abilities to induce or inhibit host cell apoptosis depending on its interaction with host cells under different conditions.

Mechanisms of the caspase-independent cell death appear to be complex. It has been shown that the Bcl-2 family protein, Bax, is involved in *Chlamydia* induced apoptosis, which is activated through a caspase-independent pathway [21], [30]. In addition, translocation of AIF from mitochondria to the nucleus has been demonstrated in *Mycobacterium bovis* induced caspase-independent cell death [46]. Our results showed that NMII infection induced translocation of AIF from mitochondria into the nucleus. Moreover, the release of cytochrome *c* and the cytosol-to-mitochondrial translocation of the pore-forming protein, Bax, in NMII infected cells occurred after 24 h post infection. These data suggests that NMII induced PARP-mediated caspase-independent apoptosis through a mechanism involving cytochrome *c* release, cytosol-to-mitochondrial translocation of Bax and nuclear translocation of AIF in THP-1 monocytes. It could speculate that bacterial factors may release during intracellular infection and could be directly action on PARP to subsequently cause cell apoptosis.

It has been suggested that reactive oxygen/nitrogen (ROS/RNS) species may be involved in PARP-mediated caspase-independent cell death [22], [23]. Pro-inflammatory cytokines such as TNF-α and IL-12 are potent inducers of ROS/RNS [38]. Previous studies have demonstrated that *C. burnetii* NMII infection is able to increase production of TNF-α and IL-12 in macrophages [31], [40], [47]. Measurement of TNF-α concentration in cell supernatants by ELISA indicated that NMII infection increased TNF-α levels and neutralization of TNF-α in NMII infected cells partially blocked PARP cleavage. These data suggest that host innate immunity such as TNF-α release might be one of the upstream factors involved in NMII induced caspase-independent apoptosis.

Although apoptosis of infected cells is an important host defense mechanism in the control of intracellular microbial infections, some intracellular bacteria have been shown to utilize host cell apoptosis for their own benefit. Induction of apoptosis in macrophages and epithelial cells was required for *Chlamydia psittaci* intracellular replication [30]. Apoptosis induced by *M. tuberculosis* and *Francisella tularensis* infection was associated with intracellular mycobacterial survival rather than with killing of the bacteria [48], [49]. Antibiotic inhibition of *C. burnetii* replication resulted in the reduction of infection-induced PARP activation. This observation indicates that apoptosis triggered by NMII infection depends on intracellular *C. burnetii* replication, suggesting that bacterial factors expressed during infection contribute to NMII induced apoptosis. Continued expression of NMII-encoded factors may maintain apoptotic signaling and facilitate the further spread of *C. burnetii*.

**Figure 9:**
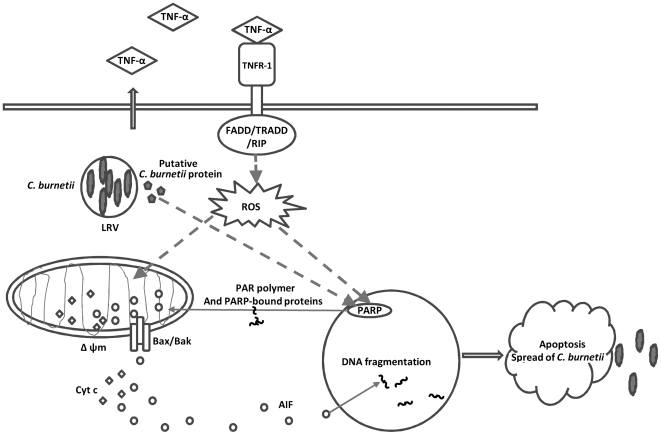
Hypothetic model of *C. burnetii* induced caspase-independent apoptosis in monocytic THP-1 cells. *C. burnetii* infection increases TNF-α release. The ligation of TNFR-1 results in recruitment of proteins containing death domains (DD), including FADD, TRADD and RIP. FADD can stimulate caspase-independent cell death through mitochondrial ROS. Bacterial factors may release during intracellular *C. burnetii* replication and may direct target on PARP that subsequently cause translocation of pore-forming protein Bax/Bak to the mitochondrial, leading to the release of AIF and cytochrome *c*. PARP activation is a sensitive indicator of DNA damage that may also be induced by oxidative stress via TNF signaling. Overactivation of PARP transduces signaling (PAR polymer and PARP-bound proteins) to cause AIF to translocate from mitochondria to the nucleus, where response for the nuclear condensation and fragmentation. PARP-mediated apoptosis result in spread of intracellular bacterial to neighboring cells.

In summary, our study demonstrated that *C. burnetii* NMII employed a mechanism to actively induce apoptosis in THP-1 cells through a caspase-independent apoptotic pathway. This is the first evidence to show a linkage between *C. burnetii* infection and caspase-independent apoptotic cell death. In addition, the results indicate that intracellular *C. burnetii* NMII replication is required for NMII induced apoptosis and suggest that maintaining a certain level of apoptosis may be important for *C. burnetii* to establish a persistent infection. Taking into account our findings, we predict a model for *C. burnetii* infection-induced caspase-independent apoptosis (Fig. 9). Future studies are needed to further identify host cell signaling molecules and bacterial factors that may be involved in *C. burnetii* induced apoptosis.
